# Monitoring Changes in the Volatile Profile of Ecuadorian Cocoa during Different Steps in Traditional On-Farm Processing

**DOI:** 10.3390/plants12223904

**Published:** 2023-11-20

**Authors:** Cyntia Yadira Erazo Solorzano, Diego Armando Tuárez García, Carlos Edison Zambrano, José Manuel Moreno-Rojas, Raquel Rodríguez Solana

**Affiliations:** 1 Faculty of Industry and Production Sciences, State Technical University of Quevedo, Av. Walter Andrade, Km 1.5 Via Santo Domingo, Quevedo 120301, Ecuador; cerazo@uteq.edu.ec (C.Y.E.S.); dtuarez@uteq.edu.ec (D.A.T.G.); 2 Faculty of Business Sciences, State Technical University of Quevedo, Av. Walter Andrade, Km 1.5 Via Santo Domingo, Quevedo 120301, Ecuador; cezambrano@uteq.edu.ec; 3 Department of Agrifood Industry and Food Quality, Andalusian Institute of Agricultural and Fisheries Research and Training (IFAPA), Alameda del Obispo, Avda. Menéndez Pidal s/n, 14004 Córdoba, Spain; 4 Foods for Health Group, Instituto Maimónides de Investigación Biomédica de Córdoba (IMIBIC), 14004 Córdoba, Spain; 5 MED–Mediterranean Institute for Agriculture, Environment and Development & CHANGE–Global Change and Sustainability Institute, Faculdade de Ciências e Tecnologia, Universidade do Algarve, Campus de Gambelas, 8005-139 Faro, Portugal

**Keywords:** *Theobroma cacao* L., fine-flavor cocoa, bulk cocoa, primary processing, volatile compounds, HS-SPME-GC–MS, chemometrics

## Abstract

The present work was conducted to evaluate the volatile profile of Ecuadorian Forastero, CCN-51, ETT103 and LR14 cocoa beans during traditional fermentation in laurel wood boxes followed by a sun-drying process. Fifty-six volatiles were identified with HS-SPME-GC–MS. Aldehydes, alcohols and ketones were the compounds that mainly characterized the fresh cocoa. The main compounds formed during the anaerobic fermentation step were esters and acids, while in the aerobic fermentation step, an increase in ester-, aldehyde- and acid-type compounds was observed. Finally, after the drying step, a notable increase in the acid (i.e., acetic acid) content was the predominant trend. According to the genotypes, ETT103 presented high contents of terpenes, alcohols, aldehydes and ketones and low contents of unfavorable acid compounds. The CCN-51 and LR14 (Trinitarian) varieties stood out for their highest amounts in acids (i.e., acetic acid) at the end of primary processing. Finally, the Forastero cocoa beans were highlighted for their low acid and high trimethylpyrazine contents. According to the chemometric and Venn diagram analyses, ETT-103 was an interestingly high-aromatic-quality variety for cocoa gourmet preparations. The results also showed the need for good control of the processing steps (using prefermentative treatments, starter cultures, etc.) on Ecuadorian genotypes of Trinitarian origin.

## 1. Introduction

Cocoa (*Theobroma cacao* L., Sterculiaceae) beans are the key raw materials in chocolate manufacturing. Among their complex composition, secondary metabolites (mainly the volatile composition) play an important role as flavoring agents and quality indicators in many cocoa-based formulations [1]. The chemical profile of the raw material depends on many factors, such as the geographical origin, including the environmental or growing conditions (e.g., soil type, water availability, sunlight and temperature), the crop season, the cocoa genotype and the postharvest stages of the cocoa (on-farm and industrial processing) [2].

There are two broad categories of cocoa beans in the world market, bulk or ordinary cocoa and fine-flavor cocoa. The first type is defined by a strong basic cocoa taste, while the second is characterized by its floral and/or fruity notes, commonly used as top-quality cocoa in gourmet products because of its exquisite taste [3,4]. Both types of cocoa are commonly cultivated in Ecuador, with the country being the main producer and worldwide exporter of this type of cocoa [5].

Raw cocoa is characterized by an astringent taste and unpleasant flavor; therefore, nonindustrialized postharvest operations or on-farm primary treatments, namely, fermentation and drying, play a critical role in the development of the flavor and aroma profile of cocoa beans [6]. Fermentation is a key cocoa-processing step, because it involves the formation of most of the flavor compounds [7]. This spontaneous process, traditionally carried out in heaps, boxes, trays or baskets, causes a series of transformations in cocoa pulp that include biochemical reactions catalyzed by a succession of indigenous microorganisms, such as yeasts and lactic and acetic acid bacteria, producing different metabolites. Thus, the concentration of important pleasant volatiles, such as some alcohols, aldehydes, ketones, esters, pyrazines and carboxylic acids, increase or are formed during fermentation [6,8,9]. Moreover, it induces pulp degradation due to the microbial strains, cotyledon death as a result of the pH and temperature changes, a reduction in astringency and bitterness (the loss of flavonoids) and color transformation (phlobaphene formation) [1]. Thus, appropriate fermentation conditions (the temperature and time, industrial or endogenous starter cultures, etc.) are crucial to the optimum formation of flavor compounds and precursors, determining the final quality of this product [10,11].

After fermentation, the beans are dried to reduce the moisture content to approximately 6–8% and to prevent mold infestations during the storage and transport of the samples [12]. This drying process continues the reactions initiated during fermentation and produces new chemical changes, resulting in flavor and color (brown) development due to cocoa bean precursors [13]. In this thermal process, the nonenzymatic browning (Maillard reaction) between reducing sugars and amino acids produces volatile fractions of pyrazines. These compounds give a roasted flavor and determine the final characteristic chocolate odor [2]. In addition, this drying process initiates major polyphenol-oxidizing reactions catalyzed by polyphenol oxidase, provides new flavor components and the loss of membrane integrity and induces a brown color formation. This fact favors the reduction in bitterness and astringency, and the development of the chocolate’s brown color in fermented cocoa beans. Moreover, the biochemical oxidation of acetic acid from the beans continues during this step [14].

In Ecuador, all these operations are mainly carried out in a traditional way by smallholders, where this crop, the fine-flavor varieties, as well the hybrid bulk variety CCN-51, are an essential source of livelihood, generating income for rural Ecuadorian families [15]. In the primary transformation of cocoa beans, there is still low levels of innovation, and ancestral production processes continue to dominate. Furthermore, despite this country being the world’s leading exporter of premium (gourmet) cocoa, there is not much information on the evolution that occurs through primary processing on the chemical composition (aromatic quality) of these appreciated and unique fine-flavor cocoa varieties.

Thus, in the present work, volatiles of the Ecuadorian fine-flavor genotypes ETT-103 and LR14 were analyzed and compared to the high-yielding and disease-resistant genotype widely cultivated in this country, the bulk CCN-51, as well as other commonly cultivated worldwide bulk cocoa beans, namely, Forastero. The study was carried out using samples of fresh cocoa beans, and with the samples submitted to traditional primary processing steps, such as fermentation in laurel wood boxes and sun-drying, to explore the chemical changes of both types of cocoa beans after different processing stages.

## 2. Results and Discussion

### 2.1. Main Changes in the Families of Volatile Compounds during Primary Processing

#### 2.1.1. Aldehydes, Alcohols and Ketones

Three families of compounds, aldehydes, alcohols and ketones, generally predominate in the fresh cocoa samples, in agreement with the literature [16]. These compounds were found in higher contents in the fine-flavor variety LR14 (Table 1).

In general, high concentrations of aldehydes and ketones, compounds conferring desirable fruity and flowery notes, are favorable for cocoa quality [6]. As can be observed in Table 1, the quantity of aldehydes tended to increase two-fold from fresh to aerobically fermented cocoa, highlighting the increase in benzene-type compounds, such as benzaldehyde with cherry, candy and almond notes and a bitter pungent perception; benzacetaldehyde with green notes and *α*-ethylidenbenzeneacetaldehyde (or 2-phenyl-2-butenal) being an important contributor to the chocolate’s aroma, with great value for the richness of the cocoa flavor, used as the main component of artificial chocolate flavors [8,17,18]. Additionally, the key marker 2-methylbutanal [19], considered a pleasant aromatic compound with malty and chocolate notes [20], showed the highest contents in the fermentation step (anaerobic fermentation in CCN-51 cocoa and aerobic fermentation in the other varieties). This result could be explained by this compound having been reported to derive from the hydrophobic free amino acid L-isoleucine (Ile) due to the formation of lactic acid bacteria (LAB) during fermentation, and its production can be improved when using certain *Saccharomyces cerevisiae* strains [21,22]. However, the concentration of the mentioned aldehydes decreased to the lowest values after the drying step, except in the case of the compound *α*-ethylidenbenzeneacetaldehyde, the only aldehyde whose concentration continued to increase, especially in the fine-flavor varieties (33% more quantity than bulk cocoa) (Table 1). On the other hand, hexanal (green notes), (E)-2-octenal (fatty and waxy notes) and 3-(methylthio)propanal (methional; potato notes) decreased or even disappeared in the last stage of fermentation. This tendency was observed in processed coffee beans [23], where the high concentrations of hexanal and (E)-2-octenal found in fresh coffee decreased until they completely disappeared during the drying process. Similarly, in the case of ketones, including 2-pentanone with fruity notes [16], 2-heptanone with fruity and floral notes [1] and 5-methyl-2-hexanone and acetophenone with musty, almond, sweet and floral notes [1,16], their contents decreased considerably with on-farm processing. Meanwhile, the opposite was observed for the hydroxy ketone acetoin (3-hydroxy-2-butanone; buttery and creamy odor), a precursor of tetramethylpyrazine, a component commonly present in chocolate products. Its concentration increased considerably following the fermentation step, as also reported in previous works (Table 1) [24].

The concentration of the family of alcohols decreased with processing (2-methylbut-3-en-2-ol, 3-methyl-2-butanol and 2-pentanol, 1-hexanol and 2-hexanol, 1-pentanol, 2-heptanol, 2-octanol and 4-methyl-5-hexen-2-ol) or increased during the fermentation (2-methyl-propanol and 2-phenylethanol) or drying (benzyl alcohol) processes (Table 1). In general, the content of the important amino-acid-derived compound 2-phenylethanol, recognized as a highly active odor key marker compound characterized by flowery, spicy and honey-like aromatic notes [25], was especially high after the aerobic fermentation and sun-drying steps, with values approximately 30 and 60 times higher, respectively, than in the initial raw cocoa (Table 1), as previously found in the literature [26]. This higher concentration after fermentation resulted from the fact that this compound is produced by yeasts via the Ehrlich pathway, where phenylalanine (Phe) is converted into 2-keto acid phenylpyruvate, which is further transformed to 2-phenylacetaldehyde and then to 2-phenylethanol [22]. This result highlights the fact that traditional Ecuadorian processing does not condition the preservation of key compounds in the aroma of cocoa.

**Table 1:** Volatile content of fresh, (anaerobic and aerobic) fermented and dried cocoa from bulk (Forastero and CCN-51) and fine-flavor (ETT-103 and LR14) varieties. Data are expressed as foldchange relative to the minimum peak area of each factor level.

	Processing					Variety				
	Fresh	Anaerobic Fermentation	Aerobic Fermentation	Drying	*p*-Value	Forastero	CCN-51	ETT103	LR14	*p*-Value
2-Methyl butanal	1.53c	2.06b	3.25a	1.00d	***	1.09	1.00	1.15	1.18	n.s.
Hexanal	6.39a	2.22b	1.00c	n.d.	***	3.33b	1.00d	4.66a	2.42c	***
(E)-2-Octenal	18.48a	1.00b	n.d.	n.d.	***	4.60b	1.00b	30.43a	4.85b	***
3-(Methylthio)propanal	1.06a	1.00a	0.00	0.00	***	1.00bc	1.97b	6.55a	0,00	***
Benzaldehyde	1.00c	1.09c	3.61a	1.85b	***	2.21b	1.00c	3.42a	3.55a	***
Benzacetaldehyde	1.33b	1.31b	3.75a	1.00c	***	1.00b	1.07b	1.68a	1.69	***
*α*-Ethylidenbenzeneacetaldehyde	1.00d	4.01c	12.08b	15.69a	***	1.74c	1.00c	7.46a	3.64b	***
∑Aldehydes	1.51b	1.49b	3.60a	1.00c	***	1.07b	1.00b	1.62a	1.51a	***
2-Methylbut-3-en-2-ol	6.52a	6.09a	3.77b	1.00c	***	1.67c	1.00d	3.27a	2.67b	***
2-Methyl-propanol	n.d.	1.00b	16.05a	n.d.	***	3.57b	12.51a	n.d.	1.00c	***
3-Methyl-2-butanol + 2-pentanol	6.23a	5.19b	1.71c	1.00c	***	1.00b	1.04b	1.48a	1.84a	**
3-Methyl-butanol	1.00c	22.05b	40.06a	35.51a	***	1.00	1.16	1.14	1.19	n.s.
2-Hexanol	5.36a	3.54b	1.96c	1.00d	***	2.16b	1.00d	1.84c	2.66a	***
2-Methyl-butanol	1.00c	7.35a	4.68b	2.08c	***	1.59b	1.00	1.04b	2.33a	**
1-Pentanol	1.64a	1.18b	1.00b	n.d.	***	3.14c	1.00d	4.49b	5.61a	***
2-Heptanol	7.20a	2.96b	1.85c	1.00d	***	3.64a	1.00c	2.70b	4.16a	***
4-Methyl-5-hexen-2-ol (Probably) ^†,††^	2.84a	1.35b	1.00c	1.27b	***	1.55a	1.00b	1.61a	1.68a	***
1-Hexanol	1.00b	1.37a	1.36a	n.d.	***	1.65a	1.00b	1.19b	1.01b	***
2-Octanol	4.14a	1.41b	1.54b	1.00b	***	3.97a	1.00b	3.13a	3.43a	***
2-Nonanol	2.35	3.35	1.00	2.10	n.s.	2.01	1.00	5.30	5.32	n.s.
*α*-Phenylethanol	1.00b	1.13b	1.57a	1.16b	***	3.52b	1.00c	3.88b	5.92a	***
Benzyl alcohol	1.00c	2.27b	2.73b	4.23a	***	1.00b	1.97a	1.11b	1.12b	***
2-Phenylethanol	1.00d	8.13c	31.66b	60.81a	***	1.23	1.34	1.04	1.00	n.s.
∑Alcohols	2.15a	1.38b	1.14c	1.00c	***	1.75b	1.00c	1.72b	2.18a	***
Acetic acid	n.d.	1.00bc	4.57b	20.95a	***	1.57	1.99	1.00	2.70	n.s.
Propanoic acid	n.d.	n.d.	n.d.	1.00a	***	n.d.	1.56a	1.00b	1.12ab	***
2-Methyl-propanoic acid	n.d.	1.00b	91.16b	883.72a	***	1.00c	2.14b	2.96ab	3.24a	**
2/3-Methyl-butanoic acid	n.d.	1.00c	75.12b	364.73a	***	1.00c	2.83b	2.70b	5.25a	***
∑Acids	n.d.	1.00c	5.44b	25.38a	***	1.21b	1.70ab	1.00b	2.45a	**
2-Pentanone	4.21a	2.28b	1.00c	1.27c	***	1.12b	1.00b	1.75a	1.73a	***
2-Heptanone + 5-methyl-2-hexanone	2.89a	1.52b	1.06c	1.00c	***	4.06b	1.00d	3.34c	5.90a	***
2-Octanone	2.14a	1.00b	1.06b	1.39b	***	4.04ab	1.00c	3.02b	4.52a	***
3-Hydroxy-2-butanone (acetoin)	n.d.	1.00b	3.59b	13.5a	***	3.46b	2.54b	1.00b	7.70a	**
2-Nonanone	2.21a	1.51ab	1.00b	2.08a	*	2.64b	1.00c	3.59b	6.13a	***
3,6-Heptanedione (probably) ^‡^	5.30a	1.79b	1.00b	1.92b	***	5.99ab	1.00c	2.09bc	3.27b	***
Acetophenone	2.42a	1.91ab	1.43bc	1.00c	**	6.80ab	1.00b	8.23a	6.97a	***
∑Ketones	2.57a	1.42b	1.00c	1.24b	***	3.28b	1.00c	3.01b	5.01a	***
Ethyl acetate	n.d.	1.00c	7.11a	1.82b	***	1.03b	3.37a	1.00b	1.16b	***
2-Pentyl acetate	n.d.	1.00b	2.75a	1.02b	***	2.85b	1.00d	1.84c	4.32a	***
2/3-Methylbutyl acetate	n.d.	1.00b	27.91a	30.62a	***	1.28	1.17	1.00	1.33	n.s.
Ethyl hexanoate	n.d.	2.61a	1.13b	1.00b	***	2.29a	1.02b	2.10a	1.00b	***
1-Methylhexyl acetate/2-heptanol acetate	n.d.	1.00b	4.93a	4.18a	***	3.80ab	1.00c	1.94bc	5.42a	**
Ethyl octanoate	n.d.	1.00c	4.85b	7.72a	***	3.80a	1.36b	1.00b	1.14b	***
Benzyl acetate	n.d.	1.00c	9.27b	34.72a	***	30.00a	10.19b	3.78c	1.00c	***
Ethyl benzeneacetate	n.d.	1.00c	14.24b	39.69a	***	2.39	1.71	1.05	1.00	n.s.
*β*-Phenylethyl acetate	n.d.	n.d.	1.00b	5.81a	***	6.86a	1.51b	1.00b	1.30b	***
Butyl benzoate	1.02b	1.00b	1.17b	1.63a	***	1.00c	1.12c	1.55b	1.82a	***
∑Esters	1.00d	28.59c	192.19a	118.59b	***	1.49b	2.14a	1.00c	1.35b	***
*β*-Myrcene	1.40	1.00	1.93	1.05	n.s.	48.35b	1.00c	100.28a	68.94ab	***
D-Limonene	1.00a	n.d.	n.d.	n.d.	***	1.00a	n.d.	n.d.	n.d.	***
Ocimene (isomers E and Z)	1.00b	1.37b	4.45a	3.23a	**	1.00c	n.d.	2.21b	3.27a	***
*γ*-Pyronene	1.00b	1.09b	3.91a	1.19b	**	1.00b	n.d.	2.10a	2.03a	**
Linalool oxide I	4.11a	2.97a	1.36b	1.00b	**	5.87bc	1.00c	29.35a	10.38b	***
Linalool oxide II	2.01a	1.39b	1.00c	1.30bc	***	1.39b	1.00b	9.33a	1.63b	***
Linalool	4.53	5.24	1.29	1.00	n.s.	10.66b	1.00b	92.76a	16.12b	***
∑Terpenes	2.14	2.12	1.51	1.00	n.s.	10.04bc	1.00c	46.61a	16.29b	***
Valerolactone	n.d.	n.d.	n.d.	1.00	***	n.d.	1.00c	1.81b	3.41a	***
Butyrolactone	n.d.	n.d.	n.d.	1.00	***	1.15b	1.00b	1.32b	3.14a	***
∑Lactones	n.d	n.d.	n.d.	1.00a	***	1.00c	1.30c	1.93b	4.22a	***
Trimethyl-pyrazine	n.d.	n.d.	n.d.	1.00a	***	5.15a	n.d.	1.00b	n.d.	***
Isophorone	n.d.	n.d.	1.00	n.d.	n.s.	1.00	n.d.	n.d.	n.d.	n.s.
Benzonitrile	n.d.	n.d.	1.00b	1.52a	***	1.00c	2.23b	2.61ab	3.07a	***
*o*-Guaiacol	n.d.	n.d.	n.d.	1.00a	***	1.00a	n.d.	n.d.	n.d.	***
∑Miscellaneous	n.d.	1.00b	18.09a	18.05a	***	6.18a	1.00b	1.50b	1.38b	***

Compounds tentatively identified according to the linear retention index calculated in previous works: 4-methyl-5-hexen-2-ol, ^†^ Costa Castro Alves et al. [27] and ^††^ Li [28]; 3,6-heptanedione, ^‡^ Raffo et al. [29]. n.d.: not detected; ns: not significant; *p*-values less than 0.05, 0.01 and 0.001 were designated with one, two or three asterisks (*), respectively. Foldchange values followed by different letters in the same row differed significantly.

#### 2.1.2. Terpenes

**Figure 1:**
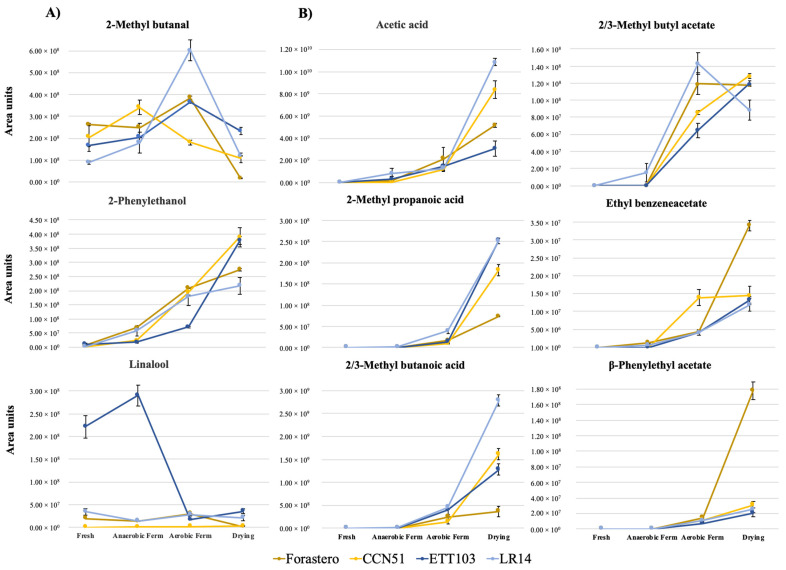
Content oscillation (area) of key aroma markers present in raw cocoa (**A**) or those that formed during the fermentation (**B**) step of the primary processing.

Terpenes are potential key floral and fruity compounds that were present at low quantities in the raw cocoa and the subsequent processed samples. However, they presented a great impact on the cocoa’s aroma due to their low perception thresholds. These compounds are major varietal aroma compounds significantly contributing to the distinctive aroma and flavor characteristics of fine-flavor cocoa [8], observing higher overall concentrations in this type of cocoa, especially in the ETT103 variety (Table 1). These findings agreed with the results obtained in the work of Ziegleder [30], who found that monoterpenes, such as the highly odor-active compound linalool (floral, rose, sweet, green and citrus notes [22]), were part of the molecules responsible for the fine-flavor in cocoa. In the present study, this compound was only strongly affected by the variety factor (*p* < 0.001), with the ETT103 variety being the most prominent in this compound (Table 1). Furthermore, it was slightly influenced by the variety × processing (*p* < 0.05) interaction, where only following the drying step was the higher content of this compound clearly differentiated in the fine-flavor varieties compared to the bulk ones (Table 2, Figure 1A). Forastero cultivars grown in Ecuador are recognized for their fine-flavor characteristics, this in part may be due to its high content of this compound. This was previously found also in Brazilian cultivars classified as Forastero, whose concentrations of linalool were higher [31].

**Table 2:** Volatile content of fresh, (anaerobic and aerobic) fermented and dried cocoa from bulk and fine-flavor varieties. Data are expressed as foldchange relative to the minimum peak area of each factor level.

	Fresh Cocoa				Anaerobic Fermentation				Aerobic Fermentation				Drying				
Compound	F	C	E	L	F	C	E	L	F	C	E	L	F	C	E	L	*p*-Value
2-Methyl butanal	19.1c	14.9cde	12.1efg	6.5h	18.1c	24.9b	14.9cde	13.1def	28.1b	13.1def	26.5b	44.0a	1.1i	8.0gh	16.9cd	8.6fgh	***
Hexanal	20.0b	8.1e	40.3a	15.3c	8.0e	1.9fg	8.0e	11.4d	8.9de	1.0fg	3.2f	n.d.	n.d.	n.d.	n.d.	n.d.	***
(E)-2-Octenal	27.6bc	n.d.	317.2a	50.6b	20.3bc	1.0c	n.d.	n.d.	n.d.	n.d.	n.d.	n.d.	n.d.	n.d.	n.d.	n.d.	***
3-(Methylthio)propanal	1.1c	4.9b	6.2b	n.d.	1.4c	n.d.	10.0a	n.d.	n.d.	n.d.	n.d.	n.d.	n.d.	n.d.	n.d.	n.d.	***
Benzaldehyde	5.0cd	1.1e	3.8cde	1.7e	3.4cde	2.0de	3.9cde	3.4cde	9.4b	3.1cde	9.6b	19.9a	1.2e	2.5cde	12.3b	5.6c	***
Benzacetaldehyde	19.0fgh	19.0fgh	31.8de	23.3efg	15.5gh	22.8efgh	24.7efg	28.7def	59.5b	47.8c	66.3b	88.9a	1.0i	12.5h	36.8d	19.7fgh	***
*α*-Ethylidenbenzeneacetaldehyde	2.2f	1.1f	4.7ef	2.8f	12.0def	3.5f	13.6def	13.8def	22.1d	11.2def	37.7c	58.3b	7.7ef	9.6def	132.9a	17.3de	***
∑Aldehydes	13.1e	10.1ef	18.3cd	10.3ef	10.8ef	13.9de	12.9e	13.6de	28.8b	19.2c	30.5b	45.2a	1.0g	6.7f	19.8c	6.8f	***
2-Methylbut-3-en-2-ol	3.0de	1.8fgh	7.1a	3.1de	2.5ef	1.8gh	6.5b	3.3cd	2.3fg	1.0i	1.6hi	3.8c	n.d.	n.d.	n.d.	2.3fg	***
2-Methyl-propanol	n.d.	n.d.	n.d.	n.d.	n.d.	n.d.	n.d.	1.0c	1.8b	6.2a	n.d.	n.d.	n.d.	n.d.	n.d.	n.d.	***
3-Methyl-2-butanol + 2-pentanol 2-Pentanol	6.1	4.7	7.7	10.5	3.4	5.6	7.2	7.9	3.4	2.7	2.2	2.6	1.1	1.1	1.0	1.4	n.s.
3-Methyl-butanol	2.0e	2.1e	1.0e	2.8e	57.7c	19.5de	32.1d	68.6c	96.1b	121.6a	35.9d	70.7c	22.0de	62.6c	133.8a	69.2c	***
2-Hexanol	16.2a	4.4f	15.2a	16.6a	7.4de	6.4e	9.5c	11.3b	6.3e	2.8fg	1.8g	8.2cd	2.7fg	1.5g	1.0g	4.3f	***
2-Methyl-butanol	n.d.	8.1cd	n.d.	n.d.	16.2ab	12.3cde	16.7ab	19.3a	15.3ab	2.7def	2.6def	17.2ab	1.0ef	2.4def	2.1def	11.2bc	**
1-Pentanol	8.4c	4.2de	23.3a	8.1cd	8.2c	1.8ef	7.2cd	14.2b	5.9cd	1.0ef	1.7ef	18.0b	n.d.	n.d.	n.d.	n.d.	***
2-Heptanol	27.7b	5.2efg	22.8c	47.9a	17.1d	6.2ef	13.0d	6.2ef	13.2d	2.2fg	2.6fg	8.5e	1.0g	2.7fg	5.5efg	5.1efg	***
4-Methyl-5-hexen-2-ol (Probably) ^†,††^	6.4ab	2.3de	5.8b	6.6a	2.3de	2.6cd	2.7cd	2.4d	2.4d	1.0f	1.5f	2.3d	1.6ef	2.2de	3.1c	2.4d	***
1-Hexanol	6.0cde	1.4g	9.0a	n.d.	6.9bcd	5.7de	4.2f	5.6de	8.0ab	5.5e	1.7g	7.1bc	n.d.	n.d.	n.d.	n.d.	***
2-Octanol	19.5a	1.0e	18.8a	21.4a	7.1c	3.1cde	6.5cd	4.1cde	12.4b	2.2cde	2.1cde	5.9cde	1.9de	4.0cde	5.0cde	3.9cde	***
2-Nonanol	11.6	1.0	24.8	24.0	5.5	5.3	51.5	25.2	11.1	6.2	1.6	7.1	5.7	4.3	11.4	33.4	n.s.
*α*-Phenylethanol	7.5de	1.0g	15.7bc	8.6de	9.6d	2.8fg	5.7ef	19.1ab	14.2c	2.0fg	13.5c	22.2a	8.2de	5.2ef	8.6de	16.5bc	***
Benzyl alcohol	1.0i	4.6fg	1.9hi	1.1i	1.9hi	12.5a	2.1hi	3.3ghi	4.2fgh	6.5def	7.4de	5.9ef	10.1abc	10.2ab	7.7cde	9.0bcd	***
2-Phenylethanol	3.5e	1.0e	6.0de	1.0e	40.0d	13.4de	19.4de	33.8de	121.2c	113.1c	41.3d	103.6c	158.6b	225.0a	218.0a	125.9bc	***
∑Alcohols	3.6b	1.1hi	3.8b	5.9a	2.7cde	1.4hi	3.0c	2.2ef	2.8cd	1.6gh	1.0i	2.3def	1.0i	1.6gh	2.1f	2.0fg	***
Acetic acid	n.d.	n.d.	n.d.	n.d.	4.8cd	1.0d	7.7cd	20.1cd	54.2cd	28.9cd	36.9cd	32.4cd	130.3b	211.8a	76.8bc	274.9a	**
Propanoic acid	n.d.	n.d.	n.d.	n.d.	n.d.	n.d.	n.d.	n.d.	n.d.	n.d.	n.d.	n.d.	n.d.	1.8a	1.0b	1.0b	***
2-Methyl-propanoic acid	n.d.	n.d.	n.d.	n.d.	n.d.	n.d.	n.d.	1.0d	22.3d	12.6d	18.6d	52.6cd	99.6c	247.9b	343.5a	341.3a	***
2/3-Methyl-butanoic acid	n.d.	n.d.	n.d.	n.d.	n.d.	n.d.	n.d.	1.0d	17.1cd	9.6cd	26.8c	31.3c	25.3c	109.8b	87.3b	189.7a	***
∑Acids	n.d.	n.d.	n.d.	n.d.	4.8e	1.1e	7.7e	20.6e	61.0de	32.6e	47.1de	44.9e	141.5c	257.3b	115.7cd	351.7a	**
2-Pentanone	4.8c	4.3c	13.7a	10.4b	3.8cde	4.8c	5.1c	4.2cd	2.7def	1.3fg	1.7fg	2.3fg	2.4efg	1.9fg	1.0g	4.6c	***
2-Heptanone + 5-methyl-2-hexanone 5-Methyl-2-hexanone	18.6b	3.1gh	9.7de	31.4a	11.1cde	3.5gh	13.6c	4.7fg	9.1e	1.0h	3.3gh	9.4e	1.0h	2.1gh	6.2f	12.4cd	***
2-Octanone	10.3a	1.1f	9.5ab	12.4a	6.1bcd	1.6ef	5.3cde	2.6def	8.7abc	1.0f	1.5ef	5.2cde	2.8def	3.2def	4.6def	11.0a	**
3-Hydroxy-2-butanone (acetoin)	n.d.	n.d.	n.d.	n.d.	n.d.	1.0	n.d.	23.1	9.2	18.5	16.8	41.8	93.1	55.8	12.8	163.1	n.s.
2-Nonanone	8.0bc	1.0d	6.9bcd	23.5a	3.6bcd	3.0bcd	17.7a	2.3bcd	9.3b	1.1cd	1.1cd	6.4bcd	2.9bcd	4.2bcd	6.8bcd	23.0a	***
3,6-Heptanedione (probably) ^‡^	31.3	3.9	13.5	25.1	13.6	2.1	6.9	2.4	9.4	1.0	1.9	2.9	13.3	4.1	1.1	7.9	n.s.
Acetophenone	16.6ab	1.1fg	20.8a	12.0de	9.2d	1.4efg	15.2abc	10.4cd	11.0bcd	1.5efg	5.0efg	11.9bcd	1.0g	1.5efg	7.1def	9.3d	***
∑Ketones	5.6b	1.0gh	3.9d	9.6a	3.3de	1.3g	4.8c	1.8fg	3.1e	0.5h	1.1gh	3.1e	1.1gh	1.1gh	2.1f	5.4bc	***
Ethyl acetate	n.d.	n.d.	n.d.	n.d.	3.1de	n.d.	1.2ef	4.7d	10.9b	37.7a	7.3c	8.0c	n.d.	8.2c	5.1d	3.1de	***
2-Pentyl acetate	n.d.	n.d.	n.d.	n.d.	6.8f	1.0gh	4.4fg	30.5bc	23.9d	19.3e	33.0b	41.2a	27.2cd	n.d.	n.d.	16.2e	***
2/3-Methylbutyl acetate	n.d.	n.d.	n.d.	n.d.	n.d.	n.d.	n.d.	1.0d	8.4ab	6.0c	4.6c	10.1a	8.3b	9.1ab	8.4ab	6.2c	***
Ethyl hexanoate	n.d.	n.d.	n.d.	n.d.	5.3b	1.0e	8.5a	5.9b	5.5b	2.1d	1.3e	n.d.	2.6cd	2.8c	2.5cd	n.d.	***
1-Methylhexyl acetate/2-heptanol acetate	n.d.	n.d.	n.d.	n.d.	29.7c	1.0c	42.8c	38.4c	272.6ab	33.8c	59.9c	186.1b	51.6c	58.2c	77.4c	281.8a	**
Ethyl octanoate	n.d.	n.d.	n.d.	n.d.	4.8gh	1.0h	4.5gh	7.2fg	33.3b	23.1c	11.4ef	14.5de	81.5a	19.6cd	15.7de	14.2de	***
Benzyl acetate	n.d.	n.d.	n.d.	n.d.	n.d.	n.d.	n.d.	1.0de	2.6cde	3.4cd	4.1c	n.d.	30.2a	7.7b	n.d.	n.d.	***
Ethyl benzeneacetate	n.d.	n.d.	n.d.	n.d.	1.7d	n.d.	n.d.	1.0d	5.7cd	18.5b	5.5cd	5.5cd	45.4a	19.3b	17.6bc	15.8bc	**
*β*-Phenylethyl acetate	n.d.	n.d.	n.d.	n.d.	n.d.	n.d.	n.d.	n.d.	1.7de	1.3e	1.0ef	1.3e	20.8a	3.6b	2.4cd	3.0bc	***
Butyl benzoate	1.3j	2.9gh	5.2cde	5.5cd	1.0ij	2.2hij	8.1a	2.7gh	2.5gh	3.8efg	2.3hi	7.8ab	8.2a	4.8def	3.4fgh	6.3bc	***
∑Esters	1.0h	2.4gh	4.2gh	4.4gh	94.5f	4.3gh	54.0fg	191.3e	490.3b	1055.3a	315.6d	453.9bc	434.7c	405.8c	309.8d	277.2d	***
*β*-Myrcene	33.8de	n.d.	176.0a	20.8de	23.1de	n.d.	126.1abc	15.3de	137.8abc	1.8e	15.4de	162.4ab	1.0e	2.3e	88.3bcd	80.7cd	**
D-Limonene	1.0a	n.d.	n.d.	n.d.	n.d.	n.d.	n.d.	n.d.	n.d.	n.d.	n.d.	n.d.	n.d.	n.d.	n.d.	n.d.	***
Ocimene (isomers E and Z)	1.8d	n.d.	7.7cd	2.3d	1.0d	n.d.	13.4bc	1.7d	15.4bc	n.d.	2.4d	34.4a	n.d.	n.d.	16.6bc	21.3b	***
*γ*-Pyronene	3.1c	n.d.	9.0bc	2.5c	1.0c	n.d.	13.6b	1.4c	16.5b	n.d.	2.9c	37.7a	n.d.	n.d.	17.3b	n.d.	***
Linalool oxide I	19.2bc	1.0c	141.8a	45.1b	13.7bc	1.3.c	116.3a	18.0bc	26.7bc	1.2c	9.3bc	31.3bc	n.d.	6.8c	31.9bc	11.5bc	***
Linalool oxide II	2.1efgh	1.1h	30.5a	1.0gh	1.9fgh	1.3gh	18.4b	2.1efgh	5.0de	1.8fgh	5.6d	4.7def	1.2gh	3.2defgh	13.9c	4.1defg	***
Linalool	31.7b	1.0b	364.8a	56.3b	22.4b	1.1b	477.4a	23.3b	50.1b	3.8b	28.2b	46.8b	2.5b	4.4b	58.5b	35.1b	*
∑Terpenes	19.1bc	1.0c	161.7a	23.1bc	12.8c	1.2c	176.5a	12.3c	54.5bc	2.6c	16.2bc	71.3b	1.6c	4.0c	53.7bc	36.1bc	***
*γ*-Valerolactone	n.d.	n.d.	n.d.	n.d.	n.d.	n.d.	n.d.	n.d.	n.d.	n.d.	n.d.	n.d.	n.d.	1.1c	1.9b	3.6a	***
Butyrolactone	n.d.	n.d.	n.d.	n.d.	n.d.	n.d.	n.d.	n.d.	n.d.	n.d.	n.d.	n.d.	1.2bc	1.0c	1.4b	3.3a	***
∑Lactones	n.d.	n.d.	n.d.	n.d.	n.d.	n.d.	n.d.	n.d.	n.d.	n.d.	n.d.	n.d.	1.0d	1.5c	2.2b	4.8a	***
Trimethyl-pyrazine	n.d.	n.d.	n.d.	n.d.	n.d.	n.d.	n.d.	n.d.	n.d.	n.d.	n.d.	n.d.	4.1a	n.d.	1.0b	n.d.	***
Isophorone	n.d.	n.d.	n.d.	n.d.	n.d.	n.d.	n.d.	n.d.	1.0	n.d.	n.d.	n.d.	n.d.	n.d.	n.d.	n.d.	n.s.
Benzonitrile	n.d.	n.d.	n.d.	n.d.	2.9ef	1.8f	1.0f	1.0f	8.1cd	9.9bc	5.7de	12.6b	n.d.	12.9b	22.1a	20.2a	***
*o*-Guaiacol	n.d.	n.d.	n.d.	n.d.	n.d.	n.d.	n.d.	n.d.	n.d.	n.d.	n.d.	n.d.	1.0a	n.d.	n.d.	n.d.	***
∑Miscellaneous	n.d.	n.d.	n.d.	n.d.	2.9d	1.8d	1.0d	1.0d	92.2a	9.9cd	5.7d	12.6cd	56.6b	12.9cd	30.3c	20.2cd	***

Compounds tentatively identified according to the linear retention index calculated in previous works: 4-methyl-5-hexen-2-ol, ^†^ Costa Castro Alves et al. [27] and ^††^ Li [28]; 3,6-heptanedione, ^‡^ Raffo et al. [29]. F: Forastero; C: CCN-51; E: ETT-103; L: LR-14. n.d.: not detected; ns: not significant; *p*-values less than 0.05, 0.01 and 0.001 were designated with one, two or three asterisks (*), respectively. Foldchange values followed by different letters in the same row differed significantly.

#### 2.1.3. Acids

Acid compounds were formed at the beginning of the fermentation process, while their concentration increased during the drying process (Table 1).

The formation of these compounds could be explained by the conversion of alcohols into other organic compounds such as lactic and acetic acids due to the growth and activity of the corresponding bacteria (lactic and acetic) [21], justifying the observed increase in the acid family and decrease in alcohols. This tendency for the acid content to increase was consistent with the individual off-odor key marker acetic and 2-methyl propanoic acids (Table 1). Acetic acid is an important odor-active compound formed in fermented beans, giving a vinegar and sour-like aroma. This compound was formed through the biochemical synthesis of ethanol oxidation during the first stage of fermentation [11], but the concentration considerably increased (20-fold) after the drying step compared to the level at the beginning of fermentation (Table 1, Figure 1B). The compounds 2-methyl propanoic acid (isobutyric acid, with rancid, buttery, cheesy and hammy notes [16]), and 3-methyl butanoic acid (sweaty aroma) could have formed (or increased in concentration) due to the aerobic putrefactive bacteria from leucine (Leu) and valine (Val), respectively [32]. In the present study, its formation started during the anaerobic phase, but as expected, their concentration in the aerobic phase stood out, with the highest contents in the dried samples (Table 1).

Despite the high concentrations of acids related to over-fermentation, with hammy and putrid off-flavor notes, the undesirable increase observed in this traditional processing is naturally unavoidable even with the implementation of improved production practices [33]. The search for varieties less susceptible to the formation of these compounds could also be a good option to improve the final quality of cocoa-based products. Thus, lower contents, mainly of acetic acid (the most abundant and limiting in the quality of cocoa), were observed in cocoa samples of bulk and ETT-103 varieties.

#### 2.1.4. Esters

Esters are an important family of aromatic compounds positively correlated with fruity notes and with a great impact in the final processed cocoa samples. These compounds are generated through the reaction of an organic acid with an alcohol during the anaerobic phase of fermentation (yeast metabolism) [34]. In the present work, the concentration of esters showed a clear increase during consecutive processing stages, namely, fermentation and drying (mainly benzene derivatives, such as benzyl acetate, ethyl benzeneacetate and *β*-phenylethyl acetate), with a varietal dependent tendency and an influence on the interaction of both factors (Table 1 and Table 2). In general, higher concentrations of the key marker odorant 2-phenylethyl acetate, a compound formed from the esterification of 2-phenylethanol [35], could be beneficial/desirable in obtaining cocoa with an improved pleasant aromatic quality [6] due to its flowery (rose) and honey-like notes [25]. This compound showed remarkable amounts in the Forastero bulk variety (Figure 1B). The higher content of this odorant compound may contribute to the aforementioned fine-flavor characteristics of this Forastero cultivar grown in Ecuador.

#### 2.1.5. Pyrazines

The family of pyrazine compounds is the result of the metabolic product of *Bacillus* genus (*B. megatrium* for trimethylpyrazine and *B. subtilis* for tetramethylpyrazine) during fermentation. Another reaction capable of producing this kind of methyl pyrazines is the condensation between the sugar breakdown products 3-hydroxy-2-butanone (acetoin), 2,3-butanediol and pyruvaldehyde and amino acids [36]. The presence of this family of compounds in fermented dried beans is an indicator of an adequate fermentation process, and predicts the quality of the beans; however, the use of endogenous yeasts and the roasting process lead to a lower pyrazine content [1]. In the present work, the only pyrazine found, trimethyl, was present in the last on-farm processing step (Table 1). This result could be attributed to the spontaneous fermentation conditions used with endogenous yeast, making this fact a subject matter for future research.

### 2.2. Chemometric Analysis of the Volatile Content Evolution along the Processing of Cocoa: Venn Diagrams and PCA

In order to find an overview of the results obtained during the traditional on-farm processing in an easier way, Venn diagrams and PCA were constructed showing the distribution of volatile compounds according to the varietal source in the different stages of on-farm or primary processing. 

**Figure 2:**
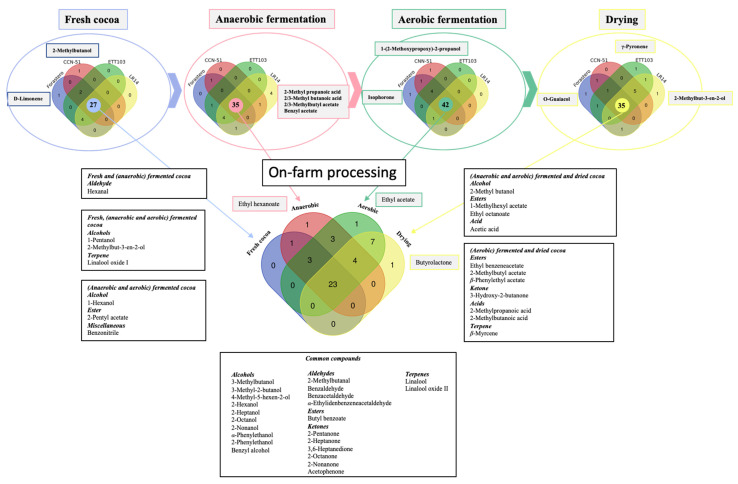
Venn diagrams of volatile compounds present in fresh, (anaerobic and aerobic) fermented and sun-dried cocoa samples from Ecuadorian bulk (Forastero and CCN-51) and fine-flavor (ETT-103 and LR14) varieties.

The Venn diagrams (Figure 2) showed the volatile profiles of fresh, fermented and dried Ecuadorian bulk (Forastero and CCN-51) and fine-flavor (ETT103 and LR14) cocoa samples. As expected, the fresh (or raw) cocoa samples had the simplest volatile profile (number of identified compounds), totaling 27 compounds common to the four studied cocoa varieties. A significant number of these compounds (23), including alcohols, aldehydes, terpenes, ketones and an ester, appeared in all the stages of the processing. The number of common volatiles increased after the anaerobic fermentation (35), with the maximum number occurring after the aerobic fermentation, with a total of 42 compounds. During both fermentation steps, the formation of certain alcohols (mainly in the anaerobic fermentation step), esters and acids (aerobic fermentation) was the most remarkable trend. Finally, the common number of volatiles decreased after drying (35). During the whole primary processing, it should be noted that the compounds common to all genotypes that could differentiate between anaerobic and aerobic fermentation and drying stages were ethyl hexanoate, ethyl acetate and butyrolactone, respectively.

**Figure 3:**
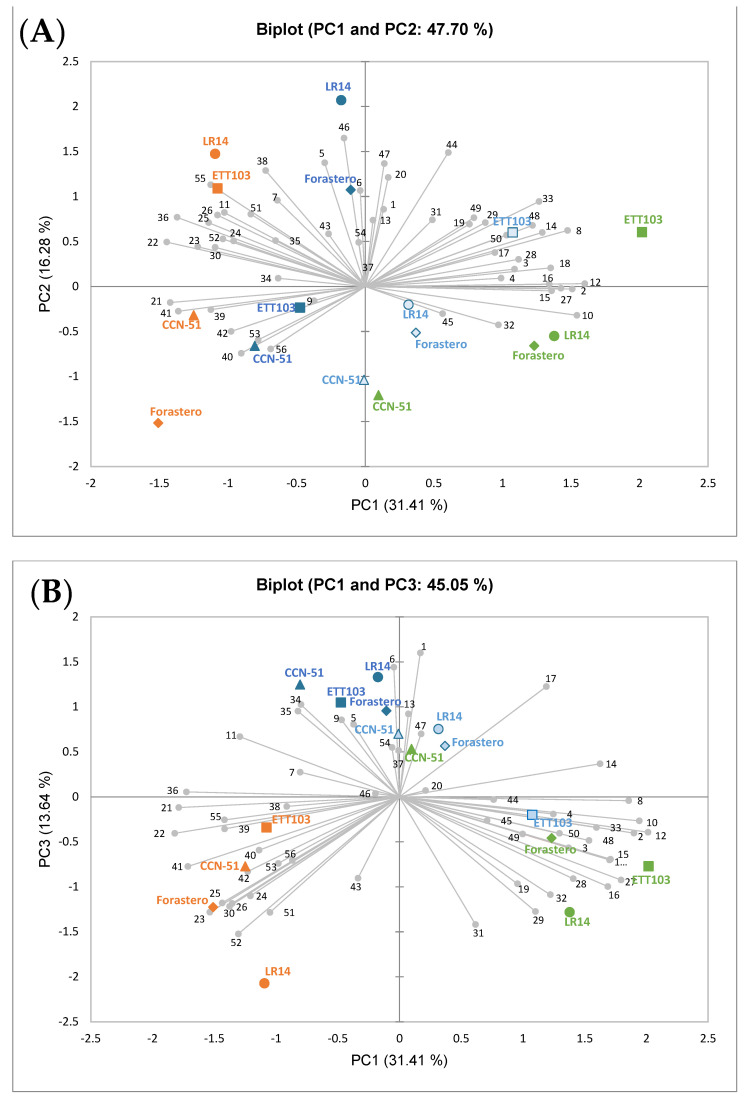
Score and loading biplots from the principal component analysis of PC1 and PC2 (**A**) and PC1 and PC3 (**B**) of the volatile profile (Table S1) obtained from the fresh (green) Ecuadorian bulk (Forastero (rhombus), CCN-51 (triangle)), fine-flavor (ETT103 (square), LR14 (circle)) cocoa and samples submitted to primary processing (anaerobic (light blue) and aerobic (dark blue) fermentation and drying (orange)).

In order to observe the overall effects of the different processing methods on the volatile composition of the cocoa beans, a principal component analysis (PCA) was performed on the total dataset. PC1, PC2 and PC3 accounted for 31.41%, 16.28% and 13.64% of the total variances, respectively, with a cumulative percentage of the total variation of 61.34%. Figure 3A,B show the scores and loading biplots defined by the first two PCs and the first and third PCs, respectively.

In Figure 3A, the PC1 axis shows a clear evolution of samples from fresh (positive values) to dried cocoa (negative values), while, in PC2, differences were observed according to the varietal origin. It is important to note that CCN-51 cocoa stood out for the slight changes in its volatile profile throughout processing in comparison with the rest of the studied varieties. On the other hand, the other three varieties were strongly affected by the aerobic fermentation process, a stage that conditioned their final on-farm (after drying) volatile profile.

The fresh cocoa samples and those obtained from anaerobic fermentation, both positioned on the positive part of PC1 (first and fourth quadrants), presented a similar volatile profile. According to the varietal origin, it should be noted that the ETT103 sample presented a more differentiated profile (first quadrant), characterized by its contents in the alcohol 2-methylbut-3-en-2-ol, the ketone acetophenone and the terpene linalool oxide I. LR14 and Forastero, located on the fourth quadrant, presented a closer profile, while CCN-51, despite also being in the same quadrant, was also more distinguished from the other two mentioned cocoa samples. However, both fine-flavor cocoa and Forastero, located in the most positive part of PC1, were characterized by higher contents in the alcohols 2-hexanol, 3-methyl-2-butanol + 2-pentanol and 2-methylbut-3-en-2-ol, the aldehyde hexanal and the ketone 2-pentanone.

Samples after aerobic fermentation showed an important change in the volatile profiles closer to the dried samples (both processed cocoa placed in the negative PC1, second and third quadrants). Despite the differences in the volatile profile of the fresh samples and the different behaviors shown throughout the anaerobic and aerobic fermentation stages, at the end of the on-farm processing, that is, after the drying step, the cocoa samples were grouped into two categories of cocoa established in the industry, bulk and fine-flavor. Thus, dried bulk cocoa, positioned in the most negative values of PC2, was characterized by high contents in the alcohols benzyl alcohol and 2-phenylethanol and the esters 2/3-methylbutyl acetate and ethyl benzeneacetate, in the Forastero variety highlighting its content in benzyl acetate and *β*-phenylethylacetate. In addition, located at high positive PC2 values, the fine-flavor varieties were notable for the contents in the terpenes ocimene (isomers E and Z), *γ*-pyronene and *β*-myrcene, the aldehydes benzaldehyde, benzacetaldehyde and *α*-ethylidenbenzeneacetaldehyde and the ester 1-methylhexyl acetate/2-heptanol acetate.

Meanwhile, Figure 3B allowed us to present information on the volatiles that generally defined each of the stages evaluated in this on-farm processing. Thus, the fresh cocoa samples, located at positive values of PC1, stood out for their contents in the alcohols 3-methyl-2-butanol + 2-pentanol and 2-hexanol, the aldehyde hexanal and the ketone 2-pentanone. As for the fermented samples, positioned at positive values of PC3, they were characterized by their contents in the aldehydes 2-methylbutanal and benzacetaldehyde, the alcohol 1-hexanol and the esters ethyl acetate and 2-pentyl acetate. Finally, the dried samples located at negative values of PC1 and PC3 (third quadrant) were defined by the content in the alcohols benzyl alcohol and 2-phenylethanol, the acids acetic acid, 2-methyl propanoic acid, 2/3-methylbutanoic acid and propanoic acid, the ketone acetoin, the ester 2/3-methylbutyl acetate and ethyl benzeneacetate, butyrolactone and benzonitrile.

## 3. Materials and Methods

### 3.1. Plant Material

Four genotypes of *Theobroma cacao* L., two bulk and two fine flavors, were used in this study. Bulk cocoa: Forastero (Iquitos Mixes Calabacillo IMC-67; Amazonian origin), and Trinitarian Colección Castro Naranjal 51 (CCN-51; (Imperial College Selection ICS-95 × IMC-67) × Canelo origin). Fine-flavor cocoa: ETT103 (National × Venezolano Amarillo type) and La Represa LR14 (Trinitarian × National). Ripe healthy cocoa beans were collected during winter (December 2019 to February 2020) from the Germplasm Bank Finca La Represa at the State Technical University of Quevedo (Quevedo, Los Ríos Province, Ecuador). The geographical location was 1°03′18″ south latitude and 79°25′24″ west longitude, at a height of 90 m above sea level.

### 3.2. Popular Ecuadorian Methods of Fermentation and Drying

The spontaneous fermentation process was carried out inside a greenhouse with the following conditions: room temperature (approx. 27 °C) and relative humidity of approx. 83%. The healthy and ripe harvested cocoa pods were opened and the beans were extracted. Raw beans were placed inside laurel wood boxes enclosed with banana leaves. The wood boxes consisted of cells (0.15 × 0.15 × 0.40 m high) 5 kg in capacity with perforations at the bottom that allowed to eliminate the mucilaginous material from cocoa beans. The cocoa beans were left to ferment for 4–5 days in the case of ETT-103 cocoa and 6–7 days for the other samples. Samples at the end of anaerobic (mucilage exudate) and aerobic (temperature drop and embryo death) fermentation were collected for analysis. During aerobic fermentation, the samples were stirred every 24 h with the aim of achieving a homogeneous fermentation.

Fermented samples were spread onto a flat concrete surface to undergo sun-drying. During the hours of exposure to the sun, the samples were stirred every three hours to obtain uniform drying. At night, the samples were collected and left to ventilate on a table located inside a greenhouse. The process lasted for approximately 6 days, until the bean humidity reached below 6.5% [37].

At each stage, a total of 200 g of cocoa samples was collected in plastic bags that were vacuum sealed and stored at −80°C until the chemical analysis.

### 3.3. Sample Preparation and HS-SPME Extraction Conditions

A total of two grams of deshelled grinded (Sammic cutter SK-3 food processor (Sevilla, Spain)) fresh, (anaerobic and aerobic) fermented or dried cocoa beans was weighed and placed in a 10 mL solid-phase microextraction (SPME) vial. The vials were placed in a CombiPal autosampler tray (CTC Analytics, Zwingen, Switzerland) provided with a support for SPME. An 80 μm divinylbenzene/carbon wide range/polydimethylsiloxane (DVB/C-WR/PDMS) 50/30 mm SPME fiber (Agilent technologies, CH, Switzerland) was selected according to previous experience [37] and used for the extraction of volatiles from cocoa beans via headspace solid-phase microextraction (HS)-SPME. Vials with cocoa samples were submitted to an equilibrium process (250 rpm and 50 °C for 5 min) followed by extraction process (250 rpm and 50 °C for 45 min) before the GC–MS injection.

### 3.4. GC–MS Instrumental Parameters

The conditions for the gas chromatography–mass spectrometry (GC–MS) technique were adapted from a previous method developed by Rodriguez-Campos et al. [6]. This process was performed in a Trace GC Ultra gas chromatograph coupled to an ISQ Single Quadrupole MS spectrometer (Thermo Fisher Scientific, Austin, TX, USA). The injection was performed in splitless mode, with the desorption time and temperature set to 10 min and 250 °C, respectively. An HP-FFAP column of 50 m × 0.32 mm and 0.50 µm film thickness (SGE Analytical Science, UK) was used for the separation. The carrier gas was helium, supplied at a constant flow rate of 1.7 mL/min. The oven temperature program was set to 40 °C for 5 min, raised to 200 °C at 10 °C/min and held for 15 min. The MS operated in electron ionization mode at 70 eV and used the selected-ion-extraction (SIE) mode to record the complete spectra and exclude undesirable masses for quantification purposes. The transfer line and source temperature of the MS were set at 230 °C and 200 °C, respectively. The tentative identification of the volatile compounds was carried out by matching the mass spectrum and retention index of the identified compounds with those from the National Institute of Standards and Technology (NIST) mass spectral library [38], in accordance with the proposed minimum reporting criteria defined by the metabolomic standard initiative [39]. Semiquantification (fold changes in relative ion peak areas) was reported for the different compounds identified.

### 3.5. Statistical Analysis

Levene’s and Shapiro–Wilk’s tests were used to, respectively, check the normality and heteroscedasticity of the data, and the variables that failed these parametric assumptions were box-cox transformed. Differences in the dataset related with the two factors studied (processing and variety) were assessed by means of a two-way analysis of variance (ANOVA), followed by an LSD all-pairwise comparison test at 5% using Statistix 10 (Analytical Software, Tallahassee, FL, USA). Thus, statistically significant differences were considered at a *p*-value ≤ 0.05. Since the data were given as peak areas with a high range of variation, the results of the ANOVA test were expressed as fold-change to an easier interpretation.

To evaluate the influence of the processing on the volatile composition of the bulk and fine-flavor varieties, independent Venn diagrams (for the fresh cocoa and for (anaerobic and aerobic) fermented and dried samples) were performed. Furthermore, a multivariate approach using a principal component analysis (PCA, XLSTAT version 2022.4.1.1375) was performed to study the interactions among the volatile compounds according to the different varietal cocoa processed samples.

## 4. Conclusions

The results showed that during primary processing, an evolution was observed for the different volatile compounds studied in a variety-dependent way. In the fermentation step, the content of ketones decreased, and the content of aldehydes, esters and acids increased or they were formed. Furthermore, a generalized reduction in the compounds was observed in the drying step, at the expense of the acids and benzyl-type acetates that increased considerably, with trimethylpyrazine being formed. The fine-flavor genotypes showed higher contents in favorable aroma compounds (aldehydes, alcohols, acetates benzyl-type and terpenes), highlighting the ETT-103 genotype for the elaboration of high-quality cocoa products.

According to the chemometric approach (PCA) and the Venn diagram analysis, the results showed a strong transformation in the volatile profiles in the aerobic fermentation step. Thus, the Forastero and LR14 varieties showed a closed profile until aerobic fermentation. However, the genotypes industrially categorized as fine-flavor (LR14 and ETT103) and bulk (CCN-51 and Forastero) cocoa only established themselves (analogous volatile profile) at the end of on-farm processing (drying step). CCN-51 was the variety that suffered the least in terms of evolution in the volatile profile during the studied processing. This variety, together with LR-14, presented a high content of undesirable acid-type compounds, indicating the need for greater control during primary processing. This fact could be due to their common Trinitarian origin.

## Data Availability

Data are contained within the article and Supplementary Materials.

## Supplementary Materials

The following supporting information can be downloaded at: https://www.mdpi.com/article/10.3390/plants12223904/s1, Table S1: volatile compounds identified in fresh, (anaerobic and aerobic) fermented and dried bulk (Forastero, F; CCN51, C) and fine-flavor (ETT103, E; LR14, L) cocoa, with the corresponding retention index (experimental and theoretical (NIST)) and the selected quantification ion; Table S2: two-way ANOVA results (*p* values) for main effects and interactions of processing (fresh, anaerobic and aerobic fermentations and drying) and varieties (Forastero, CCN-51, ETT-103 and LR-14) on the volatile profiles of cocoa samples.

## References

[1] Aprotosoaie A.C., Luca S.V., Miron A. (2016). flavor chemistry of cocoa and cocoa products-An overview. Compr. Rev. Food Sci. Food Saf..

[2] Kongor J.E., Hinneh M., de Walle D.V., Afoakwa E.O., Boeckx P., Dewettinck K. (2016). Factors influencing quality variation in cocoa (*Theobroma cacao*) bean flavour profile—A review. Food Res. Int..

[3] Quelal-Vásconez M.A., Lerma-García M.J., Pérez-Esteve É., Talens P., Barat J.M. (2020). Roadmap of cocoa quality and authenticity control in the industry: A review of conventional and alternative methods. Compr. Rev. Food Sci. Food Saf..

[4] Michel S., Baraka L.F., Ibañez A.J., Mansurova M. (2021). Mass spectrometry-based flavor monitoring of Peruvian chocolate fabrication process. Metabolites.

[5] Rottiers H., Tzompa Sosa D.A., Lemarcq V., De Winne A., De Wever J., Everaert H., Bonilla Jaime J.A., Dewettinck K., Messens K. (2019). A multipronged flavor comparison of Ecuadorian CCN51 and Nacional cocoa cultivars. Eur. Food Res. Technol..

[6] Rodriguez-Campos J., Escalona-Buendía H.B., Contreras-Ramos S.M., Orozco-Avila I., Jaramillo-Flores E., Lugo-Cervantes E. (2012). Effect of fermentation time and drying temperature on volatile compounds in cocoa. Food Chem..

[7] Schwan R.F., Wheals A.E. (2004). The microbiology of cocoa fermentation and its role in chocolate quality. Crit. Rev. Food Sci. Nutr..

[8] Cevallos-Cevallos J.M., Gysel L., Maridueña-Zavala M.G., Molina-Miranda M.J. (2018). Time-related changes in volatile compounds during fermentation of bulk and fine-flavor cocoa *(Theobroma cacao)* beans. J. Food Qual..

[9] Febrianto N.A., Zhu F. (2020). Changes in the composition of methylxanthines, polyphenols, and volatiles and sensory profiles of cocoa beans from the sul 1 genotype affected by fermentation. J. Agric. Food Chem..

[10] Biehl B., Voigt J. Biochemistry of cocoa flavour precursors. Proceedings of the 12th International Cocoa Research Conference.

[11] Sarbu I., Csutak O., Grumezescu A.M., Holban A.M. (2019). The microbiology of cocoa fermentation. Caffeinated and Cocoa Based Beverages.

[12] Nair K.P., Nair K.P. (2021). Cocoa (*Theobroma cacao* L.). Tree Crops.

[13] Kyi T.M., Daud W.R.W., Mohammad A.B., Samsudin M.W., Kadhum A.A.H., Talib M.Z.M. (2005). The kinetics of polyphenol degradation during the drying of Malaysian cocoa beans. Int. J. Food Sci. Technol..

[14] Afoakwa E.O. (2010). Chocolate Science and Technology.

[15] Díaz-Montenegro J. (2019). Livelihood Strategies and Risk Behavior of Cacao Producers in Ecuador: Effects of National Policies to Support Cacao Farmers and Specialty Cacao Landraces. Ph.D. Thesis.

[16] Rodriguez-Campos J., Escalona-Buendía H.B., Orozco-Avila I., Lugo-Cervantes E., Jaramillo-Flores M.E. (2011). Dynamics of volatile and non-volatile compounds in cocoa (*Theobroma cacao* L.) during fermentation and drying processes using principal components analysis. Food Res. Int..

[17] Fadel H.H.M., Abdel Mageed M.A., Abdel Samad A.K.M.E., Lotfy S.N. (2006). Cocoa substitute: Evaluation of sensory qualities and flavour stability. Eur. Food Res. Technol..

[18] Ziegleder G., Biehl B., Linskens H.F., Jackson J.F. (1988). Analysis of cocoa flavour components and flavour precursors. Modern Methods of Plant Analysis.

[19] Frauendorfer F., Schieberle P. (2006). Identification of the key aroma compounds in cocoa powder based on molecular sensory correlations. J. Agric. Food Chem..

[20] Batista N.N., Ramos C.L., Dias D.R., Pinheiro A.C.M., Schwan R.F. (2016). The impact of yeast starter cultures on the microbial communities and volatile compounds in cocoa fermentation and the resulting sensory attributes of chocolate. J. Food Sci. Technol..

[21] Assi-Clair B.J., Koné M.K., Kouamé K., Lahon M.C., Berthiot L., Durand N., Lebrun M., Julien-Ortiz A., Maraval I., Boulanger R. (2019). Effect of aroma potential of *Saccharomyces cerevisiae* fermentation on the volatile profile of raw cocoa and sensory attributes of chocolate produced thereof. Eur. Food Res. Technol..

[22] Rottiers H., Tzompa Sosa D.A., De Winne A., Ruales J., De Clippeleer J., De Leersnyder I., De Wever J., Everaert H., Messens K., Dewettinck K. (2019). Dynamics of volatile compounds and flavor precursors during spontaneous fermentation of fine flavor Trinitario cocoa beans. Eur. Food Res. Technol..

[23] Elhalis H., Cox J., Frank D., Zhao J. (2020). The crucial role of yeasts in the wet fermentation of coffee beans and quality. Int. J. Food Microbiol..

[24] Lee A.H., Neilson A.P., O’Keefe S.F., Ogejo J.A., Huang H., Ponder M., Chu H.S.S., Jin Q., Pilot G., Stewart A.C. (2019). A laboratory-scale model cocoa fermentation using dried, unfermented beans and artificial pulp can simulate the microbial and chemical changes of on-farm cocoa fermentation. Eur. Food Res. Technol..

[25] Calva-Estrada S.J., Utrilla-Vázquez M., Vallejo-Cardona A., Roblero-Pérez D.B., Lugo-Cervantes E. (2020). Thermal properties and volatile compounds profile of commercial dark-chocolates from different genotypes of cocoa beans (*Theobroma cacao* L.) from Latin America. Food Res. Int..

[26] Frauendorfer F., Schieberle P. (2008). Changes in key aroma compounds of criollo cocoa beans during roasting. J Agric Food Chem..

[27] Costa Castro Alves V., Flavia Azevedo da Penha M., de Oliveira Frederico Pinto N., dos Santos Garruti D. (2012). Volatile compounds profile of *Musa* FHIA 02: An option to counter losses by black sigatoka. Nat. Prod. J..

[28] Li N. (2018). Fungal Volatile Compounds: Small Molecules with Big Roles in Plant-Fungal and Fungal-Fungal Interactions. Ph.D. Thesis.

[29] Raffo A., Carcea M., Castagna C., Magrì A. (2015). Improvement of a headspace solid phase microextraction-gas chromatography/mass spectrometry method for the analysis of wheat bread volatile compounds. J. Chromatogr. A.

[30] Ziegleder G. (1990). Linalool contents as characteristic of some flavor grade cocoas. Z. Lebensm. Unters. Forch..

[31] Collin S., Fisette T., Pinto A., Souza J., Rogez H. (2023). Discriminating aroma compounds in five cocoa bean genotypes from two Brazilian states: White kerosene-like catongo, red whisky-like FL89 (Bahia), Forasteros IMC67, PA121 and P7 (Pará). Molecules.

[32] Ziegleder G., Beckett S.T., Fowler M.S., Ziegler G.R. (2009). Flavour development in cocoa and chocolate. Industrial Chocolate Manufacture and Use.

[33] Utrilla-Vázquez M., Rodríguez-Campos J., Avendaño-Arazate C.H., Gschaedler A., Lugo-Cervantes E. (2020). Analysis of volatile compounds of five varieties of Maya cocoa during fermentation and drying processes by Venn diagram and PCA. Food Res. Int..

[34] Qin X.W., Lai J.X., Tan L.H., Hao C.Y., Li F.P., He S.Z., Song Y.H. (2017). Characterization of volatile compounds in Criollo, Forastero, and Trinitario cocoa seeds (*Theobroma cacao* L.) in China. Int. J. Food Prop..

[35] Etschmann M.M.W., Sell D., Schrader J. (2005). Production of 2-phenylethanol and 2-phenylethylacetate from L-phenylalanine by coupling whole-cell biocatalysis with organophilic pervaporation. Biotechnol. Bioeng..

[36] Reineccius G.A., Keeney P.G., Weissberger W. (1972). Factors affecting the concentration of pyrazines in cocoa beans. J. Agric. Food Chem..

[37] Erazo Solorzano C.Y., Disca V., Muñoz-Redondo J.M., Tuárez García D.A., Sánchez-Parra M., Carrilo Zenteno M.D., Moreno-Rojas J.M., Rodríguez-Solana R. (2023). Effect of drying technique on the volatile content of Ecuadorian bulk and fine-flavor cocoa. Foods.

[38] Rodríguez-Solana R., Salgado J.M., Domínguez J.M., Cortés-Diéguez S. (2014). Characterization of fennel extracts and quantification of estragole: Optimization and comparison of accelerated solvent extraction and Soxhlet techniques. Ind. Crops Prod..

[39] Sumner L.W., Amberg A., Barrett D., Beale M.H., Beger R., Daykin C.A., Fan T.W.M., Fiehn O., Goodacre R., Griffin J.L. (2007). Proposed minimum reporting standards for chemical analysis: Chemical Analysis Working Group (CAWG) Metabolomics Standards Initiative (MSI). Metabolomics.

