# Supplementary material for: Monitoring Changes in the Volatile Profile of Ecuadorian Cocoa during Different Steps in Traditional On-Farm Processing
Source: Plants (Basel). 2023 Nov 20;12(22):3904. doi: 10.3390/plants12223904 (PMC10675138; doi:10.3390/plants12223904)
Supplement: Supplementary file 1 [file plants-12-03904-s001.zip › plants-2407706-supplementary.pdf]

Article

Monitoring Changes in the Volatile Profile of Ecuadorian Cocoa during Different Steps in Traditional On-Farm Processing

Cyntia Yadira Erazo Solorzano [1], Diego Armando Tuárez García [1], Carlos Edison Zambrano [2], José Manuel Moreno-Rojas [3,4,*] and Raquel Rodríguez Solana [3,5,*]

[1] Faculty of Industry and Production Sciences, State Technical University of Quevedo, Av. Walter Andrade, Km 1.5 Via Santo Domingo, Quevedo 120301, Ecuador; cerazo@uteq.edu.ec (C.Y.E.S.); dtuarez@uteq.edu.ec (D.A.T.G.)

[2] Faculty of Business Sciences, State Technical University of Quevedo, Av. Walter Andrade, Km 1.5 Via Santo Domingo, Quevedo 120301, Ecuador; cezambrano@uteq.edu.ec

[3] Department of Agrifood Industry and Food Quality, Andalusian Institute of Agricultural and Fisheries Research and Training (IFAPA), Alameda del Obispo, Avda. Menéndez Pidal s/n, 14004 Córdoba, Spain

[4] Foods for Health Group, Instituto Maimónides de Investigación Biomédica de Córdoba (IMIBIC), 14004 Córdoba, Spain

[5] MED–Mediterranean Institute for Agriculture, Environment and Development & CHANGE–Global Change and Sustainability Institute, Faculdade de Ciências e Tecnologia, Universidade do Algarve, Campus de Gambelas, 8005-139 Faro, Portugal

* Correspondence: josem.moreno.rojas@juntadeandalucia.es (J.M.M.-R.); raquel.rodriguez.solana@juntade-andalucia.es (R.R.S.)

Table S1. Volatile compounds identified in fresh, (anaerobic and aerobic) fermented, and dried bulk (Forastero and CCN51) and fine-flavour (ETT103 and LR14) cocoa, with the corresponding retention index (experimental and theoretical (NIST)) and the selected quantification ion.

	Compound	IUPAC Name	$RI_{calculated}$	RI_{NIST}	Ion
	**Aldehydes**	**Aldehydes**			
1	2-Methyl butanal	2-Methyl butanal	920	922	57
2	Hexanal	Hexanal	1087	1086	56
3	(E)-2-Octenal	(*E*)--Oct-2-enal	1443	1443	70
4	3-(Methylthio)propanal	3-Methylsulfanylpropanal	1471	1471	104
5	Benzaldehyde	Benzaldehyde	1545	1545	77
6	Benzacetaldehyde	2-Phenylacetaldehyde	1664	1663	91
7	α-Ethylidenbenzeneacetaldehyde	(*Z)*-2-Phenylbut-2-enal	1955	1961	117
		**Alcohols**			
8	2-Methylbut-3-en-2-ol	2-Methylbut-3-en-2-ol	1056	1058	71
9	2-Methyl-propanol	2-Methylpropan-1-ol	1106	1105	74
10	3-Methyl-2-butanol + 2-pentanol	3-Methylbutan-2-ol +	1131	1124/	73
11	3-Methyl-butanol	3-Methylbutan-1-ol	1213	1213	55
12	2-Hexanol	Hexan-2-ol	1226	1226	69
13	2-Methyl-butanol	2-Methylbutan-1-ol	1229	1224	57
14	1-Pentanol	Pentan-1-ol	1255	1255	55
15	2-Heptanol	Heptan-2-ol	1324	1324	55
16	4-Methyl-5-hexen-2-ol (Probably) †,††	4-Methylhex-5-en-2-ol (Probably) †, ††	1347	1344$/1348$$	81
17	1-Hexanol	Hexan-1-ol	1360	1360	56
18	2-Octanol	Octan-2-ol	1422	1422	55
19	2-Nonanol	Nonan-2-ol	1522	1521	69
20	α-Phenylethanol	1-Phenylethanol	1826	1827	79
21	Benzyl Alcohol	Phenylmethanol	1893	1893	79

22	2-Phenylethanol	2-Phenylethanol	1930	1929	91
		**Acids**			
23	Acetic acid	Acetic acid	1461	1460	60
24	Propanoic acid	Propanoic acid	1556	1556	74
25	2-Methyl-propanoic acid	2-Methylpropanoic acid	1578	1578	73
26	2/3-Methyl-butanoic acid	2/3-Methylbutanoic acid	1685	1685/1687	60
		**Ketones**			
27	2-Pentanone	Pentan-2-one	986	985	86
28	2-Heptanone + 5-methyl-2-hexanone	Heptan-2-one +	1188	1188/	58
29	2-Octanone	Octan-2-one	1290	1290	58
30	3-Hydroxy-2-butanone (acetoin)	3-Hydroxybutan-2-one (acetoin)	1295	1295	88
31	2-Nonanone	Nonan-2-one	1389	1389	58
32	3,6-Heptanedione (probably) ‡	Heptane-2,5-dione (probably) ‡	1595	1592#	57
33	Acetophenone	1-Phenylethanone	1674	1671	105
		**Esters**			
34	Ethyl acetate	Ethyl acetate	904	904	61
35	2-Pentyl acetate	Pentan-2-yl acetate	1078	1075	87
36	2/3-Methylbutyl acetate	2/3-Methylbutyl acetate	1125	1125	70
37	Ethyl hexanoate	Ethyl hexanoate	1245	1245	88
38	1-Methylhexyl acetate/2-heptanol acetate	Heptan-2-yl acetate/Heptan-2-yl acetate	1273	1266	98
39	Ethyl octanoate	Ethyl octanoate	1443	1445	88
40	Benzyl acetate	Benzyl acetate	1747	1746	108
41	Ethyl benzeneacetate	Ethyl 2-phenylacetate	1802	1800	91
42	*β*-Phenylethyl acetate	2-Phenylethyl acetate	1833	1833	104
43	Butyl benzoate	Butyl benzoate	1857	1839	105
		**Terpenes**			
44	*β*-Myrcene	7-Methyl-3-methylideneocta-1,6-diene	1161	1161	93
45	D-Limonene	(4*R*)-1-Methyl-4-prop-1-en-2-ylcyclohexene	1202	1202	68
46	Ocimene (Isomers E and Z)	3,7-Dimethylocta-1,3,6-triene (isomers E and Z)	1244	1251	93
47	*γ*-Pyronene	1,5,5-Trimethyl-6-methylidenecyclohexene	1382	1406	121
48	Linalool oxide I	6-Methyl-2-(oxiran-2-yl)hept-5-en-2-ol I	1450	1450	94
49	Linalool oxide II	6-Methyl-2-(oxiran-2-yl)hept-5-en-2-ol II	1478	1478	94
50	Linalool	3,7-Dimethylocta-1,6-dien-3-ol	1552	1552	71
		**Lactones**			
51	Valerolactone	5-Methyloxolan-2-one	1635	1629	56
52	Butyrolactone	Oxolan-2-one	1656	1651	86
		**Miscellaneous**			
53	trimethyl-Pyrazine	2,3,5-Trimethylpyrazine	1414	1414	122
54	Isophorone	3,5,5-Trimethylcyclohex-2-en-1-one	1618	1615	138
55	Benzonitrile	Benzonitrile	1629	1629	103
56	*o*-Guaiacol	2-Methoxyphenol	1881	1889	109

Compounds tentative identified according to the retention index (RI) calculated in previous works: 4-methyl-5-hexen-2-ol, † Costa Castro Alves et al. [17] and †† Li [18]; 3,6-Heptanedione, ‡ Raffo et al. [19]. RI: retention index; Ion Q: ion of quantification.

Table S2. Two way ANOVA results (*p* values) for main effects and interactions of processing (fresh, anaerobic and aerobic fermentations and drying) and varieties (Forastero, CCN-51, ETT-103 and LR-14) on the volatile profile of cocoa samples.

**Compound**	**Processin**	**Variet**	**Processing ×**	**Compound**	**Processin**	**Variet**	**Processing ×**	**Compound**	**Processin**	**Variet**	**Processing ×**
2-Methyl butanal	**<0.001**	0.088	**<0.001**	Acetic acid	**<0.001**	0.054	**0.018**	*β*-Myrcene	0.176	**<0.001**	**0.001**
Hexanal	**<0.001**	**<0.001**	**<0.001**	Propanoic acid	**<0.001**	**<0.001**	**<0.001**	D-Limonene	**<0.001**	**<0.001**	**<0.001**
(E)-2-Octenal	**<0.001**	**<0.001**	**<0.001**	2-Methyl-propanoic acid	**<0.001**	**0.001**	**<0.001**	Ocimene (E and	**0.001**	**<0.001**	**<0.001**
3-(Methylthio)propanal	**<0.001**	**<0.001**	**<0.001**	2/3-Methyl-butanoic acid	**<0.001**	**<0.001**	**<0.001**	*γ*-Pyronene	**0.001**	**0.001**	**<0.001**
Benzaldehyde	**<0.001**	**<0.001**	**<0.001**	***∑Acids***	***<0.001***	***0.009***	***0.001***	Linalool oxide I	**0.001**	**<0.001**	**<0.001**
Benzacetaldehyde	**<0.001**	**<0.001**	**<0.001**	**F-value**	74.32	5.45	5.53	Linalool oxide II	**<0.001**	**<0.001**	**<0.001**
α-	**<0.001**	**<0.001**	**<0.001**	2-Pentanone	**<0.001**	**<0.001**	**<0.001**	Linalool	0.093	**<0.001**	0.039
∑Aldehydes	***<0.001***	***<0.001***	***<0.001***	2-Heptanone + 5-Methyl-2-hexanone	**<0.001**	**<0.001**	**<0.001**	***∑Terpenes***	*0.155*	***<0.001***	***<0.001***
**F-value**	136.68	21.46	15.32	2-Octanone	**<0.001**	**<0.001**	**0.002**	**F-value**	1.99	21.76	6.54
2-Methylbut-3-en-2-ol	**<0.001**	**<0.001**	**<0.001**	3-Hydroxy-2-butanone (acetoin)	**<0.001**	**0.017**	0.081	*γ*-Valerolactone	**<0.001**	**<0.001**	**<0.001**
2-Methyl-propanol	**<0.001**	**<0.001**	**<0.001**	2-Nonanone	**0.024**	**<0.001**	**<0.001**	Butyrolactone	**<0.001**	**<0.001**	**<0.001**
3-Methyl-2-butanol + 2-Pentanol	**<0.001**	**0.001**	0.057	3,6-Heptanedione (probably)	**<0.001**	**<0.001**	0.089	***∑Lactones***	***<0.001***	***<0.001***	***<0.001***
3-Methyl-butanol	**<0.001**	0.388	**<0.001**	Acetophenone	**0.001**	**<0.001**	**<0.001**	**F-value**	500.84	59.37	59.37
2-Hexanol	**<0.001**	**<0.001**	**<0.001**	***∑Ketones***	***<0.001***	***<0.001***	***<0.001***	trimethyl-	**<0.001**	**<0.001**	**<0.001**
2-Methyl-butanol	**<0.001**	**0.002**	**0.002**	**F-value**	116.88	168.48	56.65	Isophorone	0.412	0.412	0.468
1-Pentanol	**<0.001**	**<0.001**	**<0.001**	Ethyl acetate	**<0.001**	**<0.001**	**<0.001**	Benzonitrile	**<0.001**	**<0.001**	**<0.001**
2-Heptanol	**<0.001**	**<0.001**	**<0.001**	2-Pentyl acetate	**<0.001**	**<0.001**	**<0.001**	o-Guaiacol	**<0.001**	**<0.001**	**<0.001**
4-Methyl-5-hexen-2-ol	**<0.001**	**<0.001**	**<0.001**	2/3-Methylbutyl acetate	**<0.001**	0.070	**<0.001**	***∑Miscellaneous***	***<0.001***	***<0.001***	***<0.001***
1-Hexanol	**<0.001**	**<0.001**	**<0.001**	Ethyl hexanoate	**<0.001**	**<0.001**	**<0.001**	**F-value**	19.11	15.20	6.62
2-Octanol	**<0.001**	**<0.001**	**<0.001**	1-Methylhexyl acetate/2-Heptanol	**<0.001**	**0.001**	**0.001**				
2-Nonanol	0.275	0.055	0.301	Ethyl octanoate	**<0.001**	**<0.001**	**<0.001**				
α-Phenylethanol	**<0.001**	**<0.001**	**<0.001**	Benzyl acetate	**<0.001**	**<0.001**	**<0.001**				
Benzyl alcohol	**<0.001**	**<0.001**	**<0.001**	Ethyl benzeneacetate	**<0.001**	0.059	**0.013**				
2-Phenylethanol	**<0.001**	0.066	**<0.001**	*β*-Phenylethyl acetate	**<0.001**	**<0.001**	**<0.001**				
∑Alcohols	***<0.001***	***<0.001***	***<0.001***	Butyl benzoate	**<0.001**	**<0.001**	**<0.001**				
**F-value**	97.33	63.86	34.87	***∑Esters***	***<0.001***	***<0.001***	***<0.001***				
				**F-value**	907.85	88.21	100.48				

Statistically significant effects are marked in bold ($p < 0.05$)
